# Supplementary material for: New Cysteine-Containing PEG-Glycerolipid Increases the Bloodstream Circulation Time of Upconverting Nanoparticles
Source: Molecules. 2022 Apr 26;27(9):2763. doi: 10.3390/molecules27092763 (PMC9105005; doi:10.3390/molecules27092763)
Supplement: Supplementary file 1 [file molecules-27-02763-s001.zip › molecules-1633100-supplementary.pdf]

Supplementary Information

New cysteine-containing PEG-glycerolipid increases the bloodstream circulation time of upconverting nanoparticles

M.E. Nikolaeva, A.V. Nechaev, E.V. Shmendel, R.A. Akasov, M.A. Maslov, A.F. Mironov
Lomonosov Institute of Fine Chemical Technologies, MIREA—Russian Technological University, 86 Vernadsky ave. 119571 Moscow, Russia
Federal Scientific Research Centre "Crystallography and Photonics" of RAS, 59 Leninsky ave., 119333 Moscow

The size and the zeta-potential of the nanoparticles coated with lipid **4** were evaluated using a Zetasizer Nano ZS system (Malvern Panalytica, Malvern, UK) at 25 °C in deionized water (size) and in 10 mM NaCl solution (zeta-potential).

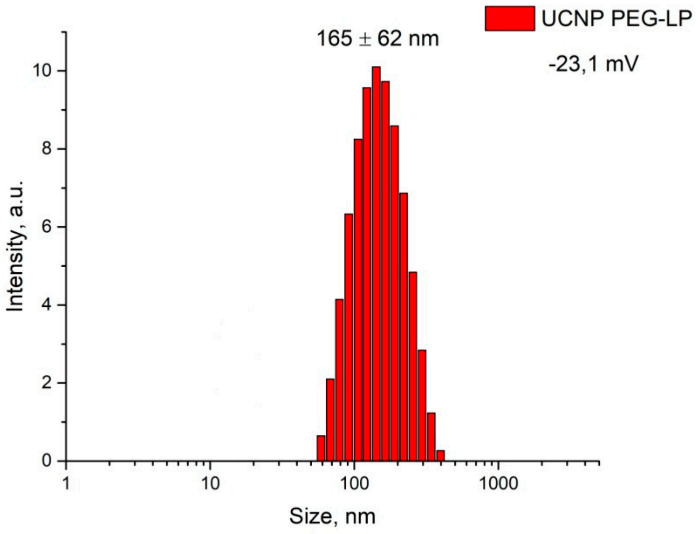

Figure S1. Size and zeta potential of nanoparticles coated with PEG-lipid **4**.
